# Vineyard dataset for automatic pruning based on main parts localization

**DOI:** 10.1016/j.dib.2025.111335

**Published:** 2025-01-27

**Authors:** Elia Pacioni, Eugenio Abengózar, Miguel Macías Macías, Carlos J. García Orellana, Horacio M. González Velasco, Antonio García Manso

**Affiliations:** a Universidad de Extremadura, Centro Universitario de Mérida, Avda. Santa Teresa de Jornet, 38. 06800 Mérida, Spain; b Universidad de Extremadura, Facultad de Ciencias, Avda. de Elvas, s/n. 06006 Badajoz, Spain; c Instituto de Computación Científica Avanzada, Avda. de la Investigación, s/n, 06006 Badajoz, Spain; d Universidad de Extremadura, Escuela Politécnica, Plaza de Caldereros, s/n. 10003 Cáceres, Spain

**Keywords:** Vineyard, Vineyard pruning, Smart agriculture, Computer vision, Object detection, Robotics

## Abstract

This dataset provides a collection of labeled images related to different parts of the vineyard (trunk, shoot, and pruned shoot), collected in Badajoz, Spain, during 2021 and 2022. The labels were created with VGG Image Annotator (VIA) software. The dataset is particularly suitable for the development of object detection models, providing a solid basis for numerous applications in smart agriculture. Considering the growing importance of precision agriculture, this data provides a valuable starting point for implementing advanced solutions. In addition, the dataset has been used to train Mask R-CNN models for precise localization of plant parts, demonstrating its value for visual processing in agricultural settings.

Specifications Table

**Table:** 

Subject	Computer Vision and Pattern Recognition
Specific subject area	*Automated agriculture, Vineyard pruning by computer vision and Robotics in agriculture*
Type of data	Image, Processed.
Data collection	Images were collected in the years 2021 and 2022. After collection, the images were manually labeled using VGG Image Annotator (VIA) [3] software.
Data source location	Location: Tierra de Barros in the southwestern province of Badajoz, Spain (Coordinates: 38.69277504545281, −6.408813985058867). Institution: Universidad de Extremadura, Instituto de Computación Científica Avanzada.
Data accessibility	Repository name: Vineyard Dataset for pruning Data identification number: 10.17632/n8cs4ns97p.2 Direct URL to data: https://data.mendeley.com/datasets/n8cs4ns97p/2 Instructions for accessing these data: Not applicable
Related research article	*None*

## 1 Value of the Data

This dataset contains vineyard images collected from 2021 to 2023 [2]. Labels were created for each image to indicate vine trunk, unpruned shoots, and pruned shoots. Thus, offering a comprehensive overview of the components of the vine.

• The data collected include photos of vineyards at different periods to provide an overview that includes the main vegetative states of the vine.
• The data can be used by researchers for computer vision projects, to build automatic pruning systems or other projects related to maintaining life. To date, no similar datasets are available, so this provides an important starting point in this area.
• The data can be used together with other datasets to form the basis of precision agriculture projects.
• The VIA format ensures compatibility, and the data can be exported to different formats.

## 2 Background

Spain's agricultural sector represents a significant portion of the nation's economy. The country's wine sector is among the most prominent in Europe, largely due to its favorable climate and extensive cultivable area, which has positioned it as a leading exporter of wine. However, one of the most pressing challenges facing the Spanish agricultural sector is the scarcity of labor, which significantly diminishes the competitiveness and profitability of farms. Considering this, numerous companies are investing in technology with the objective of compensating for the labor shortage through the implementation of intelligent automated systems. In the specific context of viticulture, pruning is a crucial practice of cutting back excess vine vegetation to contain its natural growth, thereby improving productivity and grape quality. This is traditionally done by hand during the winter months, a time when the vine is leafless and in a state of vegetative rest. Although progress has been made in mechanical pre-pruning, which serves to simplify the subsequent work of manual pruning, full automation of this process has yet to see major developments.

## 3 Data Description

The dataset consists of images (jpg format) and a json file containing labels in VIA format. Annotations represent the masks of the segmented objects.

Three categories were defined to build the dataset:

– trunk: represents the trunk of the vine, it is present in all images.
– unpruned: represents the shoots that have not yet been pruned, only the first part, the part containing the buds necessary for vine vegetation, was labeled.
– pruned: shoots that have already been pruned.

## 4 Experimental Design, Materials and Methods

### 4.1 Data

The dataset presented in this paper comprises authentic data collected annually from 2021 to 2023 in vineyards located in Terra de Barrios, located in the southwestern region of the province of Badajoz (Extremadura) in Spain. All the photographs have been taken on vines of the species “Vitis vinifera” and on the most widespread varieties in this area: “pardina” or “parda” and “macabeo” or “viura” or “lardot”.

Macabeo is one of Spain's most important white grapes and the second in extension. Pardina is a variety native to the region of Extremadura. It arises because of a process of evolution and adaptation to the characteristics of the soil and climate of Extremadura. Both varieties are used to produce white wines, but Macabeo is very popular to produce sparkling wines (cavas).

All the vines are grown on fertile irrigated land and are trained in hedgerows. They are between 10 and 20 years old and are covered by the “Ribera del Guadiana” designation of origin. They are dedicated to the intensive production of white wines exported to many countries worldwide.

The images were collected at various stages of the pruning process, which was conducted manually by qualified professionals in the field. Different devices were employed to capture the photographs, including professional cameras (model Canon EOS 2000D), and smartphones (Samsung SM-A715F). All images were captured under natural lighting and from different angles to ensure a realistic perspective.

There are a total of 536 images with different resolutions ranging from 800×600 to 6000×4000. Table 1 gives an exhaustive view of the number of images for each resolution. We chose to keep different resolutions for the images and leave it up to the users to choose which resolutions to use. Besides, having a dataset with different image resolutions can help generalization.

**Table 1:** Images sorted by resolution.

Resolution	Count
(6000, 4000)	151
(4624, 2604)	261
(2048, 1532)	23
(1600, 1197)	22
(800, 600)	79
Total	536

The color profile is always sRGB, and the f-number is 1.8. In the case of the Canon EOS2000D camera, a 34 mm lens was used.

### 4.2 Data labelling

The images were processed and labelled using VIA software to render the data suitable for use by computer vision algorithms. The labels used to annotate the images were defined based on the relevant characteristics of vines. In particular, the following categories were identified:

- Unpruned shoot (represents the first part of the unpruned shoot, generally containing the two buds needed to initiate the vegetative state)
- Pruned shoot
- Trunk

All labels are in polygon format and create the mask of the region of interest (ROI). At the conclusion of the labelling process, the accuracy of the file and the generated labels, in VIA and COCO format, was validated using ad-hoc scripts.

**Fig. 1:**
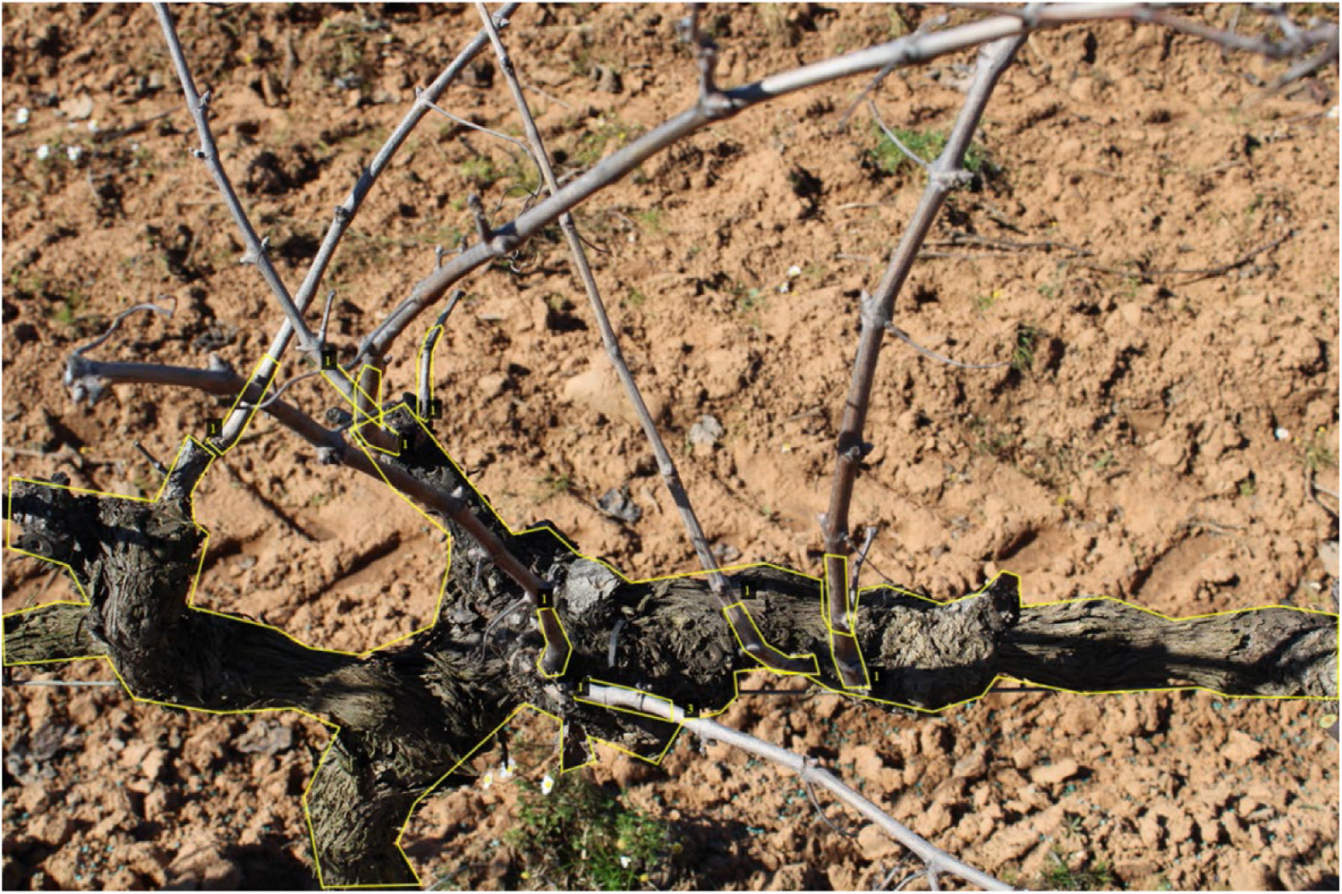
Example of a labeled dataset image.

Fig. 1 shows a labeled image presenting the trunk and shoot classes. Specifically, 1 element of the trunk class and 8 of the shoot class.

**Fig. 2:**
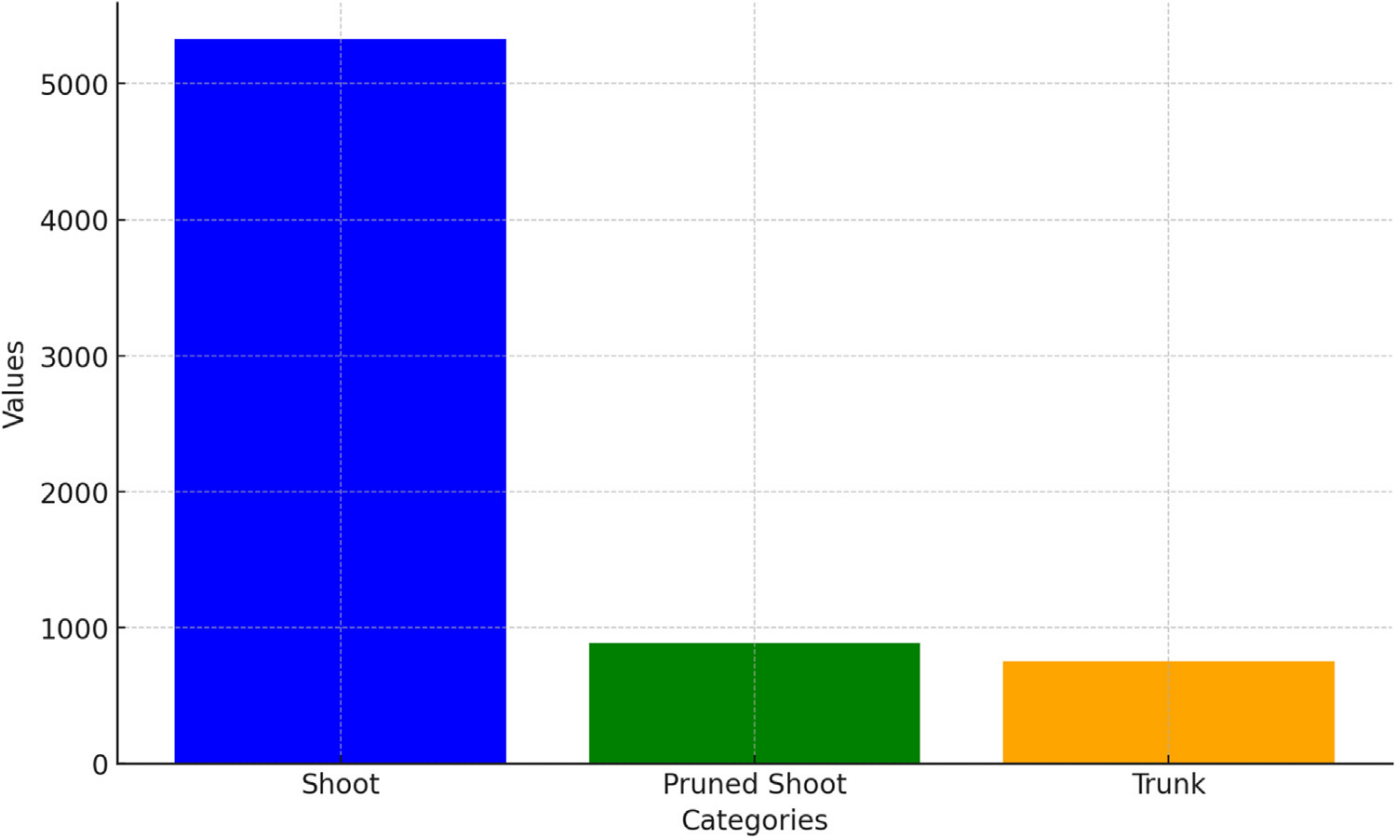
Graph of the distribution of categories shoots, pruned shoots, trunk.

Fig. 2 shows the distribution of occurrences for each category, with the most prevalent class being unpruned shoots, as this is precisely what the machine learning algorithm must focus on. Regarding the trunk, it is evident that the occurrences are less than those of the shoots, due to the influence of natural factors. Specifically, there are: 5329 shoots, 887 pruned shoots and 752 trunks, for a total of 6968 items.

### 4.3 Previous studies

This dataset has been utilized in previous studies. The authors tested the dataset with various configurations of the Mask R-CNN algorithm with the objective of developing an Automatic Vine Pruning System [1]. The obtained results are encouraging, with an mAP50 of 60.08, which allows for the satisfactory localization and segmentation of all vine components.

## Limitations

These data are limited by the accuracy of the labels. Although a rigorous process has been followed, the absence of errors in labelling cannot be guaranteed*.*

## Ethics Statement

The authors have read and follow the ethical requirements for publication in Data in Brief and confirming that the current work does not involve human subjects, animal experiments, or any data collected from social media platforms. The Data were collected by University of Extremadura staff involved in the project.

## Credit Author Statement

**Elia Pacioni:** Data Curation, Writing – Original Draft, Software, Investigation; Eugenio Abengozar-García: Data Curation, Writing – Original Draft, Investigation; **Miguel Macías Macías:** Data Curation, Conceptualization, Methodology, Supervision, Writing - Review & Editing, Funding Acquisition, **Carlos J. García Orellana:** Data Curation, Conceptualization, Supervision, Funding Acquisition **Horacio M. González Velasco:** Data Curation, Visualization, Writing - Review & Editing, **Antonio García Manso:** Resources, Supervision, Funding Acquisition.

## Data Availability

Mendeley DataVineyard dataset for pruning (Original data).
